# Genomic Profiling Reveals Distinct Routes To Complement Resistance in Klebsiella pneumoniae

**DOI:** 10.1128/IAI.00043-20

**Published:** 2020-07-21

**Authors:** Francesca L. Short, Gianna Di Sario, Nathalie T. Reichmann, Colin Kleanthous, Julian Parkhill, Peter W. Taylor

**Affiliations:** a Wellcome Sanger Institute, Wellcome Genome Campus, Hinxton, Cambridgeshire, United Kingdom; b Department of Medicine, University of Cambridge, Addenbrookes Hospital, Cambridge, United Kingdom; c School of Pharmacy, University College London, London, United Kingdom; d Department of Biochemistry, University of Oxford, Oxford, United Kingdom; e Department of Veterinary Medicine, University of Cambridge, Cambridge, United Kingdom; University of California, Davis

**Keywords:** *Klebsiella*, capsule, complement resistance, functional genomics, serum resistance, Tn-Seq, TraDIS, transposon insertion sequencing

## Abstract

The serum complement system is a first line of defense against bacterial invaders. Resistance to killing by serum enhances the capacity of Klebsiella pneumoniae to cause infection, but it is an incompletely understood virulence trait. Identifying and characterizing the factors responsible for preventing activation of, and killing by, serum complement could inform new approaches to treatment of K. pneumoniae infections. Here, we used functional genomic profiling to define the genetic basis of complement resistance in four diverse serum-resistant K. pneumoniae strains (NTUH-K2044, B5055, ATCC 43816, and RH201207), and explored their recognition by key complement components. More than 90 genes contributed to resistance in one or more strains, but only three, *rfaH*, *lpp*, and *arnD*, were common to all four strains. Deletion of the antiterminator *rfaH*, which controls the expression of capsule and O side chains, resulted in dramatic complement resistance reductions in all strains. The murein lipoprotein gene *lpp* promoted capsule retention through a mechanism dependent on its C-terminal lysine residue; its deletion led to modest reductions in complement resistance. Binding experiments with the complement components C3b and C5b-9 showed that the underlying mechanism of evasion varied in the four strains: B5055 and NTUH-K2044 appeared to bypass recognition by complement entirely, while ATCC 43816 and RH201207 were able to resist killing despite being associated with substantial levels of C5b-9. All *rfaH* and *lpp* mutants bound C3b and C5b-9 in large quantities. Our findings show that, even among this small selection of isolates, K. pneumoniae adopts differing mechanisms and utilizes distinct gene sets to avoid complement attack.

## Abstract (precis)

The serum complement system is a first line of defense against bacterial invaders. Resistance to killing by serum enhances the capacity of Klebsiella pneumoniae to cause infection, but it is an incompletely understood virulence trait. Identifying and characterizing the factors responsible for preventing activation of, and killing by, serum complement could inform new approaches to treatment of K. pneumoniae infections. Here, we used functional genomic profiling to define the genetic basis of complement resistance in four diverse serum-resistant K. pneumoniae strains (NTUH-K2044, B5055, ATCC 43816, and RH201207), and explored their recognition by key complement components.

## INTRODUCTION

The opportunistic pathogen Klebsiella pneumoniae is a major public health threat due to its propensity to become extensively drug resistant (1, 2), the emergence of hypervirulent strains (3–5), and the recent evolution and increasing prevalence of strains displaying both hypervirulence and extensive drug resistance (6, 7). Virulence in K. pneumoniae is multifactorial and depends on both core-encoded and horizontally acquired factors (8, 9). Capsule is a critical K. pneumoniae virulence determinant present in all clinical strains; mutants lacking capsule are avirulent, while overproduction of capsule is associated with hypervirulent strains and more severe disease in animal models (10, 11). More than 130 capsule locus types have been described in K. pneumoniae (12), and hypervirulent strains usually produce capsule type K1 or, less frequently, K2. Nine lipopolysaccharide (LPS) O-side-chain groups have been identified and characterized in K. pneumoniae (13); these moieties modulate innate immune signaling and may contribute to serum resistance. Horizontally acquired virulence genes include siderophores and capsule upregulators (9, 14). In general, understanding of K. pneumoniae pathogenesis is confounded by the phylogenetic breadth of infectious lineages, and by the diversity of the virulence factors themselves.

The complement system, comprising more than 20 proteins in serum and tissue fluids, is a first line of defense against bacterial invaders that have breached the host’s epithelial barriers. Resistance to complement is strongly correlated with the capacity for systemic survival, multiplication, and spread of a wide range of Gram-negative pathogens (15), and is a major virulence trait that enables K. pneumoniae to elicit invasive infections (16, 17). The complement cascade can be activated via the classical, alternative, and lectin pathways, which each act in a precise sequence of reactions to facilitate C3b deposition onto the target bacterial surface. The classical pathway is initiated following recognition of antigen-antibody complexes on the bacterial cell surface by hexameric C1q, whereas the lectin pathway begins with detection of bacterial surfaces by pattern recognition molecules such as mannose-binding lectin or the ficolins (15, 18, 19). All pathways converge at C3 cleavage, with the larger cleavage product C3b covalently bound to the target surface. Accumulation of anchored C3b by amplification leads to the assembly of C5 convertases that generate the C5b cleavage product, which spontaneously associates with one molecule each of C6, C7, and C8 and with multiple copies of C9 to form the C5b-9 membrane attack complex. In complement-susceptible bacteria, C5b-9 complexes intercalate into the outer membrane (OM) bilayer and perturb the cytoplasmic membrane through an incompletely defined process (20–22).

Gram-negative bacterial resistance to complement can be due to failure of activation of any of the complement pathways; degradation of activated complement proteins; arrest of activated pathways by complement inhibitors such as C1-inhibitor protein (C1-Inh), factor H (fH), and C4 binding protein (C4bp); or the inability of C5b-9 complexes to assemble and insert into the OM (which can be a result of impedance by bacterial surface structures) (15). The basis of the complement resistance of K. pneumoniae is still poorly understood. Although it has been reported that limiting complement activation and C3b accumulation is the primary mode of resistance, both complement-resistant and -susceptible clinical isolates and mutants may activate complement cascades after exposure to human serum (16, 23–25). Multiple different factors can influence serum resistance in K. pneumoniae, including capsule type and amount, O-antigen type, and various surface proteins; capsules and O antigens have each been invoked as the main determinant of complement resistance (9, 26). However, a recent study of >150 K. pneumoniae clinical isolates from Thailand with various complement susceptibilities concluded that susceptibility did not correlate with the presence of specific genes, particular capsule types, or even with the hypercapsulation phenotype of the isolates (27). This study highlighted the main limitation of collective studies on complement resistance in K. pneumoniae to date—that although many resistance factors are individually well characterized, there is very limited understanding of how their activities play out in different combinations or across diverse isolates of K. pneumoniae.

Untangling the mechanisms behind complement resistance in K. pneumoniae will lead to better understanding of the virulence of this bacterium and will provide avenues to target complement resistance in the clinic, particularly in view of growing interest in the targeting of capsule and other virulence factors as an anti-infective strategy for K. pneumoniae (28–30). In particular, developing generally applicable (rather than K type-specific) therapeutics that promote complement killing requires deeper knowledge of the activity of different complement resistance factors in diverse strains. In this study, we used functional genomic profiling by transposon-directed insertion site sequencing (TraDIS) to define the genetic basis of serum survival in four diverse strains of K. pneumoniae. We show that complement resistance is multifactorial and strain specific, and we identify RfaH and Lpp as shared K. pneumoniae resistance determinants. Two of the strains evaded complement killing by preventing C3b and C5b-9 accumulation at the cell surface, which was disrupted in Δ*rfaH* and Δ*lpp* mutants, while the remaining two strains were resistant to serum despite substantial C5b-9 deposition. Our results present a picture of at least two distinct modes of complement resistance in K. pneumoniae and point to RfaH and Lpp as potential targets for complement-sensitizing therapeutics.

## RESULTS

### Serum-resistant isolates of K. pneumoniae.

**FIG 1:**
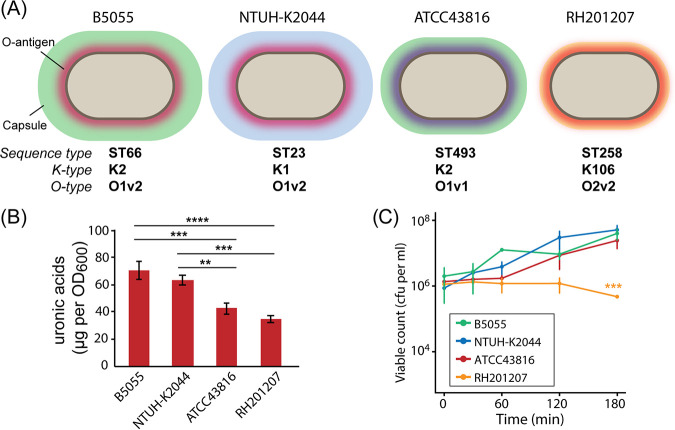
Characteristics of K. pneumoniae strains used in this study. (A) Schematic of the four strains used in this study, with sequence type, O-antigen, and capsule types indicated. (B) Quantification of capsular uronic acids in four K. pneumoniae strains (*n* = 3). Statistically significant differences between strains were determined by one-way analysis of variance (ANOVA) (overall *P* < 0.0001) followed by Tukey’s honestly significant difference (HSD) test; **, *P* < 0.01; ***, *P* < 0.001; ****, *P* < 0.0001. (C) Resistance of the four K. pneumoniae strains to killing by pooled human serum (*n* = 3). Strains were compared by two-factor repeated-measures ANOVA (overall *P* < 0.0001), and Tukey’s HSD test at *t* = 180 showed RH201207 to be significantly different from each of the other three strains; ***, *P* < 0.001.

Three well-studied hypervirulent K. pneumoniae strains and one recently isolated classical strain were tested for survival in human serum (Fig. 1A; see also Table S1 in the supplemental material). B5055 (sequence type 66 [ST66]) produces a type K2 capsule and was originally isolated from a sputum sample in the 1920s. NTUH-K2044 is a hypervirulent strain (sequence type 23) that produces a K1 capsule and was the first characterized liver abscess-causing K. pneumoniae isolate (31). ATCC 43816 is another K2 strain that is commonly used in mouse virulence studies (32). K. pneumoniae RH201207 is a colistin-resistant ST258 strain obtained from Public Health England in 2012 (33). These strains differ in their capsule production as determined by uronic acid assay, with B5055 and NTUH-K2044 producing copious capsule and ATCC 43816 and RH201207 producing less (Fig. 1B). All four strains survived exposure to 66% normal human serum (a potent source of complement) over a 3-h incubation period at 37°C when an inoculum of 1 × 10^6^ cells was employed (Fig. 1C); strains B5055, NTUH-K2044, and ATCC 43816 proliferated in serum, whereas viable counts for RH201207 did not change between 0 and 2 h but showed a slight reduction at 3 h. A sensitive control strain, Escherichia coli DH5α, showed no viability after a 30-min incubation, and killing of all strains was completely abrogated by heat inactivation of the serum (56°C, 30 min; data not shown).

### TraDIS analysis of complement resistance in K. pneumoniae isolates.

We performed transposon insertion sequencing of saturated mutant libraries exposed to serum to define the genes contributing to serum survival in each of the four K. pneumoniae strains. The K. pneumoniae B5055 library was constructed for this study by conjugative delivery of pDS1028 and contained 225,000 unique transposon insertions (see Materials and Methods and Table S2 in the supplemental material), while NTUH-K2044, ATCC 43816 and RH201207 mutant library construction has been previously described (33, 34). Our experimental strategy was similar to that used in previous work with E. coli ST131 (35), with libraries treated with either normal human serum or heat-inactivated serum for 90 min, outgrown for 2 h, and sequenced and mapped using the BioTraDIS pipeline (36). Putative serum resistance genes were defined as those with altered mutant abundance in the serum-treated libraries in comparison to that in the control libraries treated with heat-inactivated serum (normal serum versus heat-inactivated serum log_2_ fold change of less than −1 or more than 1; significance [*q* value] of <0.005; see Table S3 in the supplemental material). Comparing to the heat-inactivated serum control, rather than the input library, minimizes the chance of spurious hits to mutants with general growth defects.

**FIG 2:**
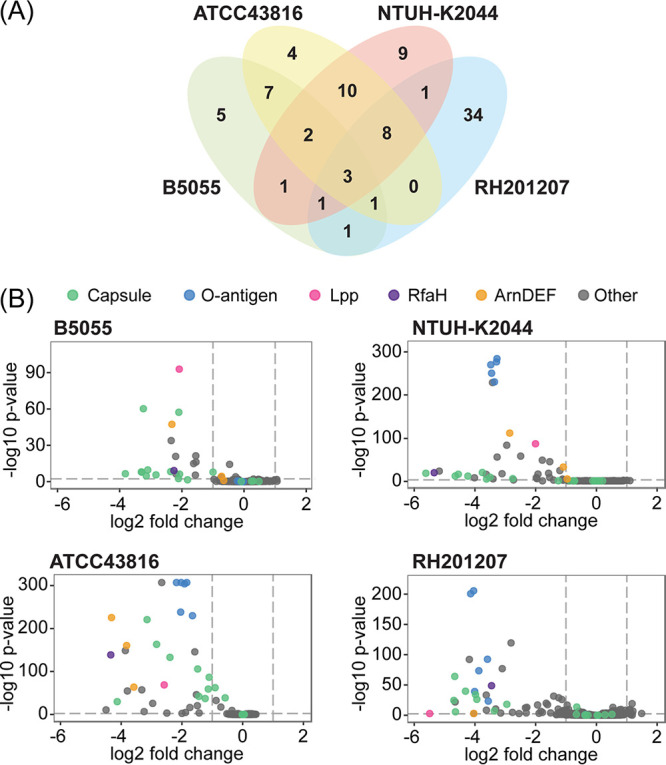
Genes contributing to serum resistance in four K. pneumoniae strains. (A) Venn diagram showing the overlap in genes involved in serum survival in each strain. Hit genes are defined as those with a log_2_ fold change (log_2_FC) of less than −1 or more than 1 (*q* value < 0.005). Full results are shown in Table S3 and TraDIS hits in Table S4 in the supplemental material. (B) Abundance of transposon mutants following serum treatment relative to that after treatment with heat-inactivated serum. Volcano plots of log_2_ fold change and log_10_ *P* value are shown for each strain. Genes with very low read counts in any condition are excluded. Key resistance factors (capsule, O antigen, Lpp, RfaH, and ArnDEF) are indicated by color.

**FIG 3:**
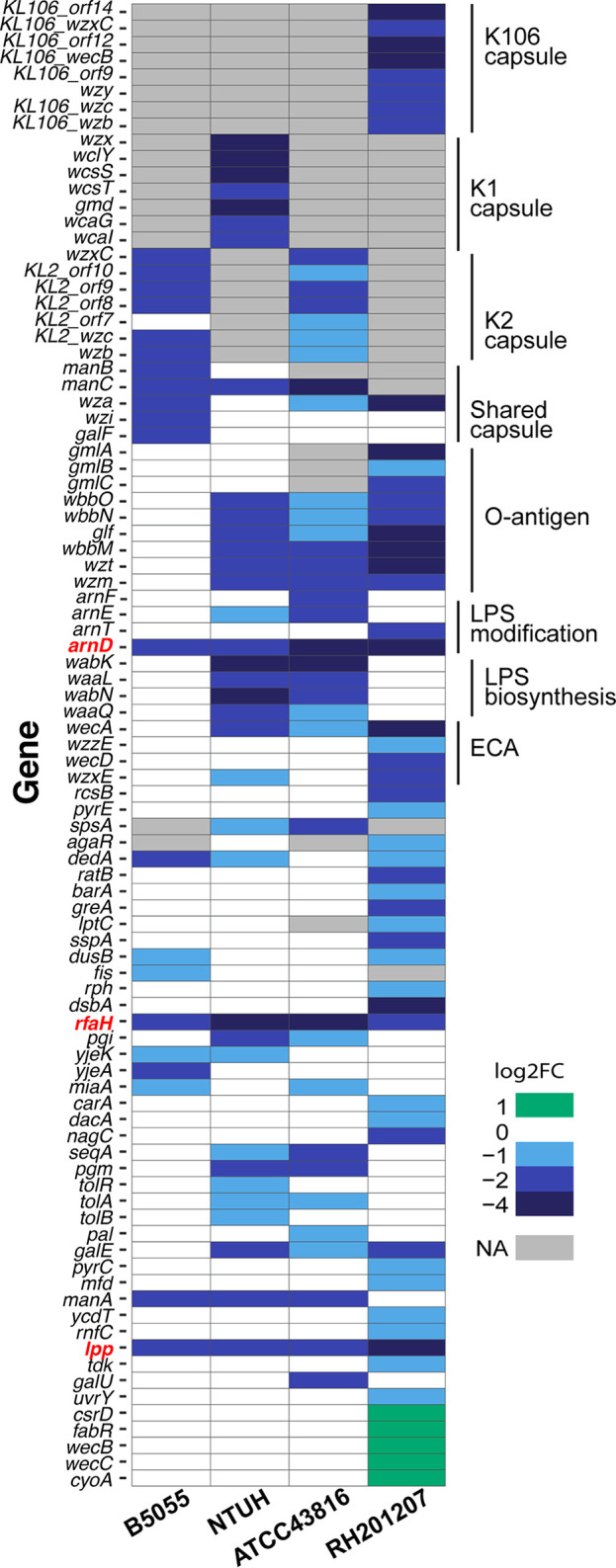
Strain specificity of complement resistance in K. pneumoniae. Discontinuous heat map of TraDIS hits for complement resistance. Homologues across different strains were determined by BLASTp search (cutoff, >90% amino acid identity) in the process of constructing the K. pneumoniae pangenome (see Materials and Methods). Capsule and O-antigen locus types were determined using Kaptive-Web, and the corresponding gene names are used. The three genes outside the capsule and LPS loci that were required for complement resistance in all four strains are indicated in red text. Genes marked “NA” are either absent from that strain or have been excluded from the comparative analysis due to having very low read counts under any condition. Full details are in Tables S3 and S4.

A total of 93 genes were identified that altered serum survival in one or more K. pneumoniae strains (Fig. 2; see also Table S4 in the supplemental material), with the number of hits in each strain ranging from 22 (for B5055) to 54 (for RH201207). These genes included 43 core or soft core K. pneumoniae genes (present in >99% or 95 to 99% of K. pneumoniae strains), 24 shell genes (15 to 95% of strains), and 26 cloud genes (<15% of strains). Despite the high proportion of shell and cloud genes, 60 of the serum survival-related genes were present in all four of the strains examined. Putative serum resistance genes came from multiple functional categories that included synthesis of surface polysaccharides, metabolism, cell surface or membrane structure and function, and transcriptional regulation (Fig. 3; and Table S4). Overall, there was an unexpected strain specificity in the exact genes identified; even among the 60 gene hits present in all four strains, the majority (35 genes) influenced serum survival in only one strain, 22 genes were hits in two or three strains, and only three genes affected serum survival in all four K. pneumoniae strains (Fig. 2A and Fig. 3).

### Contribution of capsule to serum resistance.

Capsule biosynthetic genes (*cps*) were among the putative serum survival factors in the four strains investigated. The proportion of *cps* locus genes contributing to complement resistance and the magnitude of the fitness changes involved varied between isolates (Fig. 2B and Fig. 3). Note that mutagenesis of genes of the capsule locus can cause secondary cell envelope defects (shown for *wza* and *wzi* [37]), and not all *cps* locus mutations entirely eliminate capsule production (34, 38); therefore, complete consistency of selection across all genes of the *cps* locus is not expected. With K. pneumoniae B5055, which produces copious amounts of K2 capsule, 11/18 genes of the *cps* locus were called as hits, accounting for half of the serum survival determinants of this strain. They included the exporter *wzi*, the sugar precursor genes *manBC* and *galF*, and the majority of K-type specific genes in the central operon of the K2 locus (Fig. 3; see also Fig. S1 in the supplemental material). The majority of these genes were also required in the K2 strain ATCC 43816 (Fig. 3 and Fig. S1), with the exceptions of *wzi*, and the sugar precursor genes *manB* and *ugd* (which had too few reads in this strain for serum-specific effects to be measured). In NTUH-K2044, 8/20 *cps* genes were called as hits and in RH201207 this proportion was 9/19 (Fig. 3 and Fig. S1). Because the pDS1028 transposon has transcriptional readout from one end, transposon insertions are not predicted to dramatically disrupt downstream gene expression in the NTUH-K2044, B5055, and ATCC 43816 libraries. This effect is clear in NTUH-K2044, where transposon insertions in several genes of the *cps* locus (e.g., KP1_3714, KP1_3718 through KP1_3720) are counterselected by serum on one strand but unaffected on the other (Fig. S1). The RH201207 library was constructed using a different transposon, and transcriptional read-through is not expected in this library. Our TraDIS results indicate that all K. pneumoniae strains require capsule to some extent to withstand serum challenge. Known regulators of capsule biosynthesis also influenced serum survival, including the antiterminator gene *rfaH* in all four isolates (39, 40), *rmpC* (BN49_pII0025) in B5055 only (41), and *rcsB* in RH201207 (42). We hypothesize that the *rcsB* mutant showed a serum survival defect only in the RH201207 background because this strain produces less capsule than the other three strains, making it more sensitive to mutations that further reduce capsule expression. Mutation of the *rmpC* gene had no effect in NTUH-K2044; however, this strain encodes both chromosomal and plasmid copies of *rmpC*.

### Contribution of LPS O side chains to serum resistance.

Enterobacteriaceae species lacking LPS O side chains are generally susceptible to C5b-9-mediated killing (43), and introduction of genes determining O side chains into a highly complement-sensitive rough Escherichia coli strain elicited a large increase in complement resistance (44). With our four K. pneumoniae strains, the majority of O-antigen genes showed a serum fitness defect when mutated (Fig. 2B and Fig. 3), with the exception of those of K. pneumoniae B5055. This is surprising because B5055 encodes the same O-antigen type (O1v1) as NTUH-K2044, in which O-antigen mutants showed a drastic fitness defect (Fig. 2B and Fig. 3). We suggest that the B5055 strain is almost completely protected from serum bactericidal activity by its thick K2 capsule, masking the additional protective activity of the O antigen. K. pneumoniae ATCC 43816 produces K2 capsule, albeit in smaller amounts than that produced by B5055, but still required O antigen for serum survival. These findings suggest that the K2 capsule is sufficient to completely protect from complement-mediated killing when produced in copious amounts, while the K1 capsule is not, at least in these isolates.

Lipopolysaccharide (LPS) core biosynthetic genes contributed to serum fitness in isolates ATCC 43816 and NTUH-K2044 (see Table S4 in the supplemental material), although the same genes were either essential or had no effect on resistance in B5055, and were also not identified as statistically significant hits in RH201207. Note that mutation of many LPS core genes causes a severe general fitness defect, so their specific contributions to serum resistance are not always easy to define. A small subset of the genes (*arnD* to *arnF*) in the *arn* (*pmr*) operon responsible for LPS lipid A modification showed complement resistance defects in one or more strains when mutated (Fig. 2B). This was unexpected, as the l-Ara4N lipid A modification is rarely made *in vitro* and is not produced under rich medium conditions like those used in this study (45). Loss of any of the *arnDEF* genes was previously shown to reduce K. pneumoniae mucoviscosity in a genome-wide density-based screen (34), and we suggest that reduced capsule production underpins the serum survival defects seen here.

### Other genes implicated in serum survival.

Mutation of several genes involved in cell membrane or cell wall structure and function resulted in fitness defects in serum. These genes included *dacA* (RH201207 only), which is involved in cell wall biosynthesis, the inner membrane protein *dedA* (in isolates B5055, NTUH-K2044, and ATCC 43816) which has a role in membrane integrity, and components of the *tol-pal* outer membrane transporter (NTUH-K2044 and ATCC 43816). Finally, the outer membrane lipoprotein Lpp was required for full serum resistance in all four strains. A number of metabolic genes were also implicated in serum survival, primarily those involved in pyrimidine metabolism, and in metabolism of carbohydrates (Fig. 3 and Table S4). Some of these genes (*pgi* and *pgm*) are involved in precursor molecule biosynthesis for capsule and LPS.

### Increased serum survival genes in K. pneumoniae RH201207.

Five genes of K. pneumoniae RH201207, namely *csrD*, *fabR*, *wecB*, *wecC*, and *cyoA*, led to increased serum fitness when mutated. CsrD promotes degradation of the capsule-regulating small RNA CsrB; mutation of *csrD* can promote capsule production (as measured by density) (34), which may explain the enhanced serum survival of this mutant. FabR, WecB, and WecC are not predicted to affect capsule, but all three genes have roles relating to the cell envelope: FabR is a transcriptional regulator which controls the ratio of saturated to unsaturated fatty acids in the cell membrane, and WecB and WecC produce the second component of enterobacterial common antigen (*N-*acetyl*-* d-mannosaminuronic acid) and attach this to UDP-GlcNac. We speculate that loss of *wecB* and *wecC* increases the pool of UDP-GlcNac in the cell, which is then diverted into O-antigen biosynthesis (which also utilizes UDP-GlcNac) (46). The cytochrome ubiquinol oxidase component CyoA also resulted in increased serum survival when mutated through an unknown mechanism. The identification of mutants with increased serum fitness in RH201207, but not in the other K. pneumoniae strains, is consistent with the observation that serum survival of K. pneumoniae RH201207 is less dependent on capsule.

### Confirmation of RfaH and Lpp as shared serum resistance factors in K. pneumoniae isolates.

**FIG 4:**
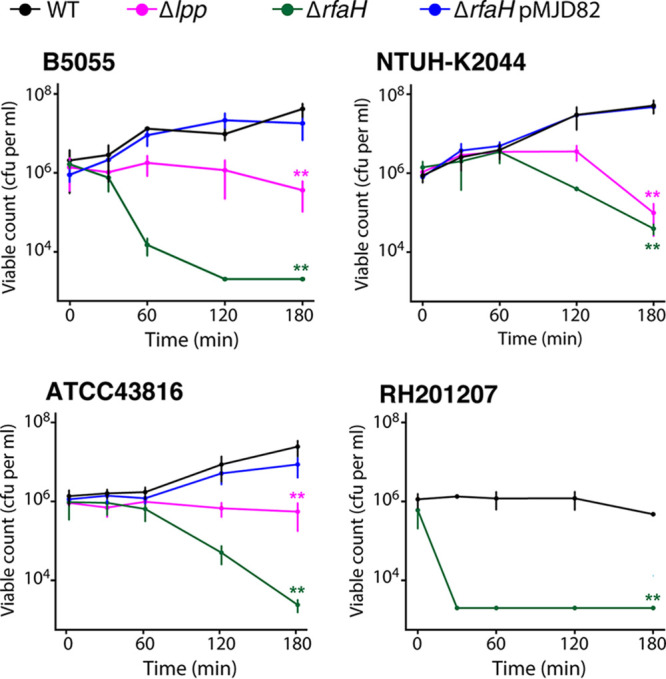
Validation of serum survival defects in Δ*rfaH* and Δ*lpp* mutants. Total bacterial viable count of K. pneumoniae strains and key mutants following incubation with 66% pooled normal human serum (see Materials and Methods). The detection limit of the assay is 2 × 10^3^ viable cells per ml. Overall statistical significance was determined by two-factor repeated measures ANOVA (*P* < 0.0001 for all strains), mutants were compared to the wild type (WT) at *t* = 180 by single-factor ANOVA and Dunnett’s test at *t* = 180 (**, *P* < 0.01). For ATCC 43816 WT and mutants, *n* = 5; for all other strains, *n* = 3.

Only the following three genes affected serum survival in all four strains tested: the LPS modification gene *arnD*, the outer membrane lipoprotein *lpp*, and the transcription antiterminator *rfaH* (Fig. 2 and 3). We selected Lpp and RfaH for further characterization as potential core serum resistance factors of K. pneumoniae. ArnD was not selected for followup because we failed to detect the relevant LPS modification *in vitro* (which is consistent with previous reports that the modification is made *in vitro* only under very specific conditions [45]) and therefore presumed its activity was indirect, though the potential role of lipid A modifications in K. pneumoniae complement resistance may be of interest for a future study. Deletion mutants of *rfaH* and *lpp* were constructed in K. pneumoniae NTUH-K2044, B5055, and ATCC 43816 by allelic exchange. In isolate RH201207, an insertion mutant in *rfaH* was obtained, but an *Δlpp* mutant could not be generated despite multiple attempts. Serum survival assays were conducted with an inoculum of 10^6^ cells in 66% normal human serum, and bacterial counts were monitored for 3 h (Fig. 4). Loss of *rfaH* caused a large reduction in serum survival in all four strains, and complementation with plasmid-encoded *rfaH* expressed from its native promoter restored wild-type survival, confirming the importance of RfaH in complement resistance (Fig. 4). Loss of *lpp* caused a modest change in complement sensitivity (Fig. 4); these mutants lost the ability to proliferate in serum (note that *lpp* disruption does not cause a general growth defect; see Fig. S2B and Table S3 in the supplemental material), and with the NTUH-K2044 Δ*lpp* mutant, delayed complement killing was observed. The *Δlpp* mutations could not be complemented by expression of *lpp* from its native promoter due to unexpected toxicity during cloning. Expression of *lpp* from an arabinose-inducible promoter also failed to complement the serum proliferation defect of the *Δlpp* mutants. We suspect that this was due to insufficient expression. In addition, proliferation of the vector-only control strains was impaired by addition of arabinose (data not shown). Although we were unable to find an appropriate system for complementation of the *Δlpp* mutants, their phenotypes align with the results of the genome-scale screens (Fig. 2 and 3), as well as with published work on Lpp in K. pneumoniae NTUH-K2044 (47).

We were intrigued by the varied serum fitness effects caused by different *cps* locus mutations seen in TraDIS, and several randomly isolated capsule locus mutants of ATC C43816 and RH201207 were also examined for serum survival in order to further validate the genome-scale screens (Fig. S2). Each of these mutants showed the phenotype predicted based on TraDIS screening, namely, ATCC 43816 i-*wcaJ*, which was not identified as a serum resistance gene, multiplied to the same extent as that of the wild type, ATCC 43816 i-*wza* did not proliferate in serum, and RH201207 i-*wzc* was rapidly susceptible. RH201207 i-*wcaJ*, which was not a hit, was viable after 90 min (our TraDIS time point) but showed a delayed susceptibility to serum. Note that *wcaJ* deletion in K. pneumoniae does not completely eliminate K2 capsule production and can also have pleiotropic effects that include rounded cell morphology and increased fitness under nutrient limitation (37, 38)—therefore, the full resistance of ATCC 43816 i-*wcaJ* does not preclude a role for capsule in the complement resistance of this strain. Taken together, the results of serum survival assays with defined mutants show perfect agreement with the phenotypes predicted from TraDIS screens (for 11/11 mutants), and establish RfaH and Lpp as shared serum resistance factors in K. pneumoniae. These experiments also revealed additional subtleties in the serum resistance phenotypes of the mutants, with survival patterns roughly following the underlying resistance of the parent strain (for example, ATCC 43816 Δ*rfaH* and RH201207 Δ*rfaH* mutants), and some differences (e.g., RH201207 i-*wcaJ*) were only revealed at later stages of incubation.

### Lpp influences capsule retention but not capsule production and requires lysine-78.

**FIG 5:**
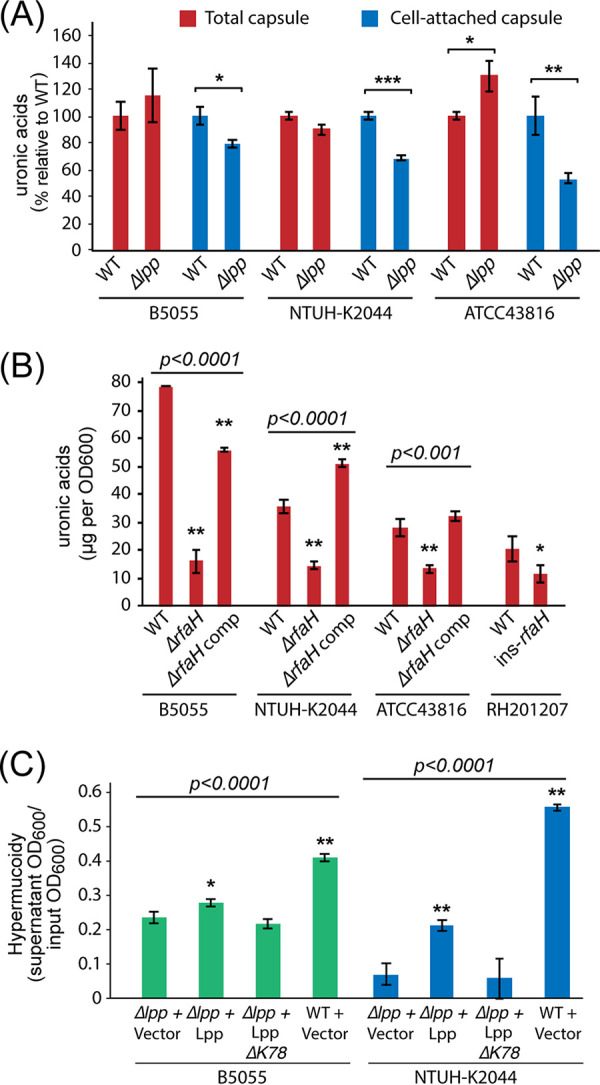
Effects of Lpp and RfaH on capsule production and retention. (A) Comparison of total and cell-attached capsule content of wild-type and Δ*lpp* mutants of K. pneumoniae ATCC 43816, B5055, and NTUH-K2044 (*n* = 3). Uronic acids were either quantified directly from culture or following a single wash and resuspension in LB (see Materials and Methods), and Δ*lpp* values were normalized to the WT from the same strain and condition. All three strains showed a significant reduction in cell-associated capsule, while total capsule was unchanged or increased (one-way ANOVA relative to WT; *, *P* < 0.05; **, *P* < 0.01; ***, *P* < 0.001). (B) Comparison of capsule production in Δ*rfaH* mutant and complemented mutant strains (*n* = 3). Overall statistical significance for each strain was determined by one-way ANOVA; the Δ*rfaH* mutant and complemented strains were compared to the WT by Dunnett’s *post hoc* test. The RH201207 Δ*rfaH* mutant was compared to the WT by one-way ANOVA. *, *P* < 0.05; **, *P* < 0.01. (C) Partial complementation of K. pneumoniae Δ*lpp* mutants using an inducible vector (*n* = 3). The hypermucoidy assay for capsule was performed on stationary-phase, arabinose-induced cultures washed once in phosphate-buffered saline (PBS). Induction of wild-type Lpp partially restored the hypermucoid phenotype of the NTUH-K2044 and B5055 Δ*lpp* mutants. This effect was not seen with the empty vector or with an Lpp construct lacking its C-terminal lysine (ΔK78). Overall significance for each strain was determined by one-way ANOVA, followed by Dunnett’s *post hoc* test to compare each WT or complemented strain to Δ*lpp* plus vector. *, *P* < 0.05; **, *P* < 0.01.

The antiterminator RfaH and the murein lipoprotein Lpp contributed to complement resistance in all four K. pneumoniae strains. Lpp is an extremely abundant protein that contributes to cell envelope integrity by connecting peptidoglycan to the cell outer membrane (47, 48). We observed that the Δ*lpp* mutant colonies were flat and unstructured in comparison to those of the wild type, although their opacity suggested that they still produced capsule. To examine the effect of the *lpp* mutation further, we measured total and cell-attached capsule using the uronic acid assay. All three K. pneumoniae Δ*lpp* mutants produced capsule at wild-type levels but showed moderate decreases in amounts of cell-associated capsule (Fig. 5A). Mutants of *rfaH* showed dramatically reduced capsule amounts (Fig. 5B). We then tested whether Lpp activity requires covalent linkage to peptidoglycan, mediated through the ε-amino group of the C-terminal lysine residue in Lpp and the meso-diaminopimelic acid residue on the peptidoglycan peptide stem (49). Expression of Lpp from an arabinose-inducible vector partially complemented the hypermucoid phenotype of NTUH-K2044 and B5055 (Fig. 5C). Partial complementation was not seen with an Lpp-ΔK78 construct, confirming that the C-terminal lysine is required in order for Lpp to promote capsule retention in both K1 and K2 strains. The shared serum survival factors Lpp and RfaH therefore both appear to function at least partly through effects on capsule.

### Deposition of C3b and C5b-9 complexes.

**FIG 6:**
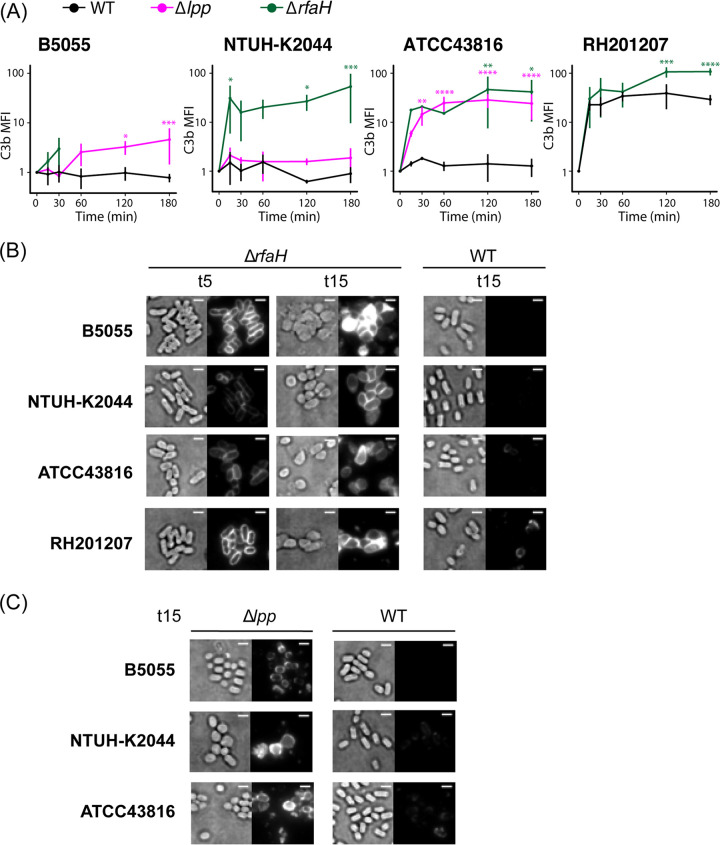
C3b binding to the bacterial cell surface. (A) Flow cytometry-based determination of C3b binding to ATCC 43816, B5055, NTUH-K2044, RH201207, and their respective mutants were measured after 15, 30, 60, 120, and 180 min of incubation in human pooled serum at 37°C (*n* = 3). Values were converted to AU, setting *T*_0_ to 1. Two-way repeated measures ANOVA with uncorrected Fisher’s least significant difference (LSD test revealed significance as follows: *, *P* ≤ 0.05; **, *P* ≤ 0.01; ***, *P* ≤ 0.001; ****, *P* ≤ 0.0001. For the B5055 Δ*rfaH* mutant, data were collected only at the first two time points due to cell lysis detected by release of cytoplasmic fluorescent marker from labeled cells. (B and C) Fluorescence microscopy of 5-min and 15-min serum-exposed Δ*rfaH* and WT cells (B) or 15-min serum exposed Δ*lpp* and WT cells (C) following incubation with APC-conjugated anti-C3b antibody. Data are representative of three independent experiments. For comparison of binding patterns and intensity, transillumination (left) and fluorescence images (right) are normalized within each panel. Bar, 2 μm.

**FIG 7:**
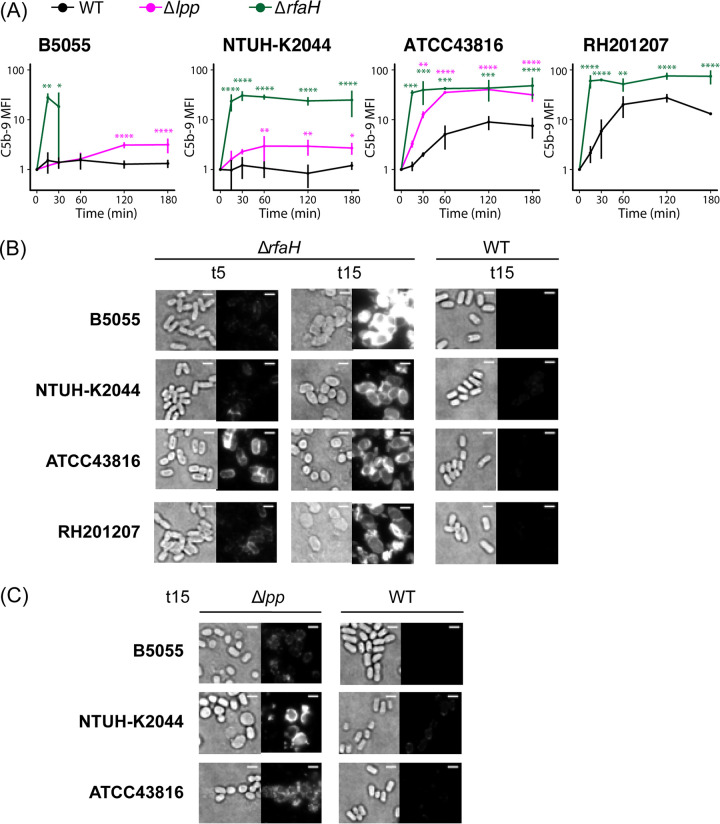
C5b-9 binding to the bacterial cell surface. (A) Flow cytometry-based determination of C5b-9 formation on ATCC 43816, B5055, NTUH-K2044, RH201207, and their respective mutants after 15, 30, 60, 120, and 180 min of incubation in human pooled serum at 37°C (*n* = 3). Values were converted to AU, setting *T*_0_ to 1. Two-way repeated-measures ANOVA with uncorrected Fisher’s LSD test revealed significance as follows: *, *P* ≤ 0.05; **, *P* ≤ 0.01; ***, *P* ≤ 0.001; ****, *P* ≤ 0.0001. For the B5055 Δ*rfaH* mutant, data were collected only at the first two time points due to cell lysis detected by release of cytoplasmic fluorescent marker from labeled cells. (B and C) Fluorescence microscopy of 5-min and 15-min serum-exposed Δ*rfaH* and WT cells (B) or 15-min serum-exposed Δ*lpp* and WT cells (C) following incubation with mouse anti-C5b-9 antibody and AF488 goat anti-mouse IgG. Data are representative of three independent experiments. For comparison of binding patterns and intensity, transillumination (left) and fluorescence images (right) are normalized within each panel. Bar, 2 μm.

Genome-scale screening revealed a very high degree of strain specificity in the serum resistance determinants across four K. pneumoniae strains. We decided to explore complement activation by these strains and how this is affected by the loss of *rfaH* or *lpp*. Activation of any or all complement pathways will lead to C3b generation and binding to the target bacterial surface; subsequent formation of C5 convertase complexes may lead to deposition of membrane attack complexes and cell death (15, 19). Surface C3b deposition and C5b-9 formation on K. pneumoniae strains and mutants during incubation with human serum are reported in Fig. 6 and 7 and in Fig. S3 and S4 in the supplemental material. The three hypervirulent strains showed little to no C3b binding, while serum exposure of RH201207 led to a considerable increase in levels of C3b and C5b-9 over time (Fig. 6A and Fig. 7A; Fig. S3 and Fig. S4A). ATCC 43816 also showed C5b-9 binding at later time points, while B5055 and NTUH-K2044 did not. In all backgrounds, the deletion of *rfaH* led to significant levels of C3b and C5b-9 binding compared to that of the wild type, with a peak after 2 to 3 h of serum exposure (Fig. 6A and Fig. 7A), confirming that the mechanism of serum killing observed (Fig. 4) is through formation of the membrane attack complex. With the B5055 Δ*rfaH* mutant, cells could not be examined after the 30 min time point due to cell lysis as determined by the release of cytoplasmic green fluorescent protein (GFP) from strain B5055 Δ*rfaH* pFLS21 (Fig. S4B). Imaging of the Δ*rfaH* mutants showed that C3b binding is evenly distributed over the cell surface and occurs within 5 min of serum exposure (Fig. 6B), while C5-9 deposition is minimal at 5 min (except for ATCC 43816) and uniformly detected at 15 min (Fig. 7B). Similarly, Δ*lpp* mutants were also found to significantly bind C3b and C5b-9 compared to the wild type, although to a lesser extent than the Δ*rfaH* mutants (Fig. 6A and Fig. 7A; Fig. S3 and S4). Most Δ*lpp* mutant cells maintained their rod shape following 15 min of serum exposure (Fig. 6C and 7C), which correlates with increased serum susceptibility only after longer exposure times (Fig. 4). By examining cell population dynamics, we observed that Δ*lpp* mutants showed a similar distribution to wild-type cells (Fig. S3 and S4A, third columns). In contrast, Δ*rfaH* mutants displayed a more compact distribution in the Q2 quadrant, suggesting that not only do more Δ*rfaH* cells bind C3b and C5b-9 over time, but that the level of binding to individual cells increases. These findings indicate that B5055, NTUH-K2044, ATCC 43816, and RH201207 activate the complement system to different extents and that loss of *lpp* or *rfaH* increases the recruitment of complement components.

## DISCUSSION

Resistance to killing by complement is an important yet incompletely understood feature of K. pneumoniae pathogenesis (4, 8, 27). The prominent polysaccharide capsule has been invoked as a key determinant of resistance by virtue of its capacity to limit C3b deposition or assembly of the membrane attack complex (8, 26), but it is clear that other factors also contribute to the complement-resistant phenotype (27). Resistance to serum killing is associated with K. pneumoniae hypervirulence, and we therefore selected three well-studied hypervirulent strains, as well as a recently isolated clinical strain, for our analyses. To our knowledge, this study represents the first multistrain functional genomics study of complement resistance in any bacterial species.

TraDIS identified 93 genes that impacted serum survival in one or more strains, but only three of these, *rfaH*, *lpp*, and *arnD*, were common to all four strains. All three genes influence the physical characteristics of the outer surface of K. pneumoniae. RfaH controls transcription of operons that direct synthesis, assembly, and export of the lipopolysaccharide core and capsular polysaccharide in E. coli and other Gram-negative bacteria (40), the abundant peptidoglycan-linked outer membrane protein Lpp is involved in the maintenance of cell envelope integrity and retention of capsule at the cell surface (Fig. 5A) (34, 49), and the *arn* operon encodes proteins that participate in the addition of 4-amino-4-deoxy-l-arabinose to lipid A (50) and may also affect capsule levels through an unknown mechanism (34). Deletion of *rfaH* markedly increased complement susceptibility in all four strains, confirming the key contributions of capsule and LPS O side chains to the resistant phenotype. However, there were strain differences in the rate of complement killing; the K. pneumoniae RH201207 Δ*rfaH* mutant was rapidly killed, while ATCC 43816 and NTUH-K2044 displayed a delayed killing response typical of smooth (O-side-chain-replete) complement-susceptible Gram-negative bacteria (51). RH201207 possesses LPS O side chains but elaborates less capsule than the other three. These complement susceptibility profiles emphasize the interdependence of the various surface structures that contribute to serum resistance. Deletion of *lpp* in the three hypervirulent isolates modified the serum responses but to various degrees. The loss of proliferation in serum of strains ATCC 43816 and B5055 was not sufficient to convert them to full complement susceptibility, whereas the degree of complement killing of the K. pneumoniae NTUH-K2044 Δ*lpp* mutant was more pronounced. Although capsule retention is impaired in the *lpp* mutants, reducing the protective barrier against complement binding, the presence of large amounts of unattached polysaccharide is likely to have caused off-target complement activation (25) and depletion of complement components in the serum, resulting in less pronounced killing than that in *rfaH* mutants.

Removal of the capsule by deletion of *rfaH* (Fig. 5B) led to significant deposition of C3b on the outer surface in all four strain backgrounds. Deletion of *rfaH* presumably caused loss of O side chains as well as capsule, as shown in E. coli and other Gram-negative bacteria, including Salmonella enterica and Yersinia enterocolitica (40, 52, 53). The formation of C5b-9 complexes at the cell surface and subsequent changes in cell morphology point to a loss in integrity of both the outer membrane and peptidoglycan layer, eventually leading to cell lysis, although the exact mechanism by which the inner membrane is disrupted is not yet understood. With the Δ*lpp* mutants, which have detached capsule (Fig. 5) and increased membrane permeability but retain their O antigen (47), sufficient deposition of C3b and perturbation of the cell envelope by C5b-9 complexes occurred to prevent proliferation in serum, as seen in Fig. 4. While B5055 and NTUH-K2044 did not show detectable C3b or C5b-9 levels by flow cytometry, the complement-resistant ATCC 43816 strain showed a limited increase in levels of C5b-9 complexes following serum exposure, despite these not functioning as bactericidal entities (Fig. 6A and Fig. 7A; Fig. S4A). Finally, although the classical isolate RH201207 survived 2- to 3-h serum incubation, both C3b and C5b-9 levels rose dramatically following incubation with serum.

These differences in the interplay between surface factors and the complement system are unlikely to be due to differences in strain-to-strain gene content. Around half of the hit genes were present in all four strains but contributed to complement resistance in only one or two (46 of 93 total genes, 60 of which were present in all strains), and this trend held when the classical RH201207 strain was excluded (of 36 hits in B5055-NTUH-K2044-ATCC 43816 shared genes, 12 were specific to one strain, 19 were hits for two strains, and only 5 were hits for 3 strains). However, the degree of strain specificity we found is broadly comparable to that observed for daptomycin resistance genes in two strains of Streptococcus pneumoniae, which showed only 50% overlap despite the two strains sharing 85% of their genes (54). Furthermore, bacteria such as Salmonella spp., Mycobacterium tuberculosis, and Pseudomonas aeruginosa have been shown by TraDIS/transposon insertion sequencing (Tn-Seq) methods to possess strain-specific essential gene sets (55–57).

Strain-specific effects are likely to be due to a combination of imperfect hit identification, functional divergence of genes in different strains, and context-dependent fitness contributions of genes with the same activity, due to either redundancy with other factors or differences in the relative contribution of each gene to overall bacterial surface architecture. For example, the O1v2-type O antigen produced by both NTUH-K2044 and B5055 contributed to serum resistance only in the former strain, presumably because in B5055 the protection from the capsule is so strong that other factors are not needed. We speculate that such context-dependent fitness effects may be a common feature in bacterial populations. Our finding that vastly different gene sets underpin serum survival in four strains supports the notion that serum resistance is determined by the overall biophysical properties of the cell surface, rather than by any single factor, and also shows that there are multiple routes by which a complement-resistant cell surface can be generated.

A limitation of our study is that K. pneumoniae is a highly genetically diverse species (58), and the four isolates that we studied do not cover the range of potential combinations of cell surface structures that may impact survival in serum. We did note that the classical strain RH201207 was markedly different from the three hypervirulent strains in terms of genes involved in complement resistance and complement binding patterns; it would be useful to explore the properties of other classical strains in future studies. Another limitation is that in order to maintain library diversity and provide enough material for sequencing, we based our TraDIS strategy on that used by Phan and coworkers (35), employing a library inoculum of 10^8^ CFU with only a single 90-min time point, which may have missed delayed or subtle effects on complement resistance. Although high-throughput mutagenesis studies such as ours are the only way to profile the contributions of all nonessential genes to serum survival, mutation or deletion of genes encoding major surface structures (such as capsule, LPS O side chains and abundant membrane proteins) may force a major reconfiguration of the cell surface as a compensatory mechanism to deal with envelope stress (59); conversion of complement-resistant cells to complement-susceptible cells could be caused by this compensatory response, rather than by loss of the structure itself. In particular, capsule locus mutations can have a range of secondary effects that include changes to cell envelope integrity, cell morphology, and growth rate, and some do not fully abolish capsule production (37, 38). Such effects cannot be detected or avoided by employing different mutagenesis strategies (e.g., gene deletion versus transposon insertion) or by complementation. While these findings fit with the range of serum resistance phenotypes that we observed among different *cps* locus mutants (Fig. S1 and Fig. S2A), they also suggest that any data derived from capsule mutant strains should be interpreted with caution. More direct information comes from recent studies using phage-derived capsule depolymerases, where K. pneumoniae strains are stripped of capsule prior to treatment with serum. In this way, at least eight different capsular types of K. pneumoniae have been confirmed to protect from serum to date, including type K1 (of NTUH-K2044) (60–65). The magnitude of the change in serum sensitivity following enzymatic capsule removal varies depending on both the strain and the capsule type. We are currently examining the impact of capsule removal on complement susceptibility in systematic fashion using enzymes selective for the most frequently isolated K. pneumoniae capsular serotypes. Despite its inherent limitations, our genome-scale screening gives a picture consistent with recent phage depolymerase work and collective molecular microbiological studies (9, 26)—K. pneumoniae capsule can protect from serum killing, and the strength of this protection depends on capsule type, capsule thickness, and the strain background.

The data suggest that K. pneumoniae may adopt different strategies for evasion of complement-mediated attack. Isolates may fail to strongly activate complement pathways (B5055 and NTUH-K2044) or may activate one or more pathways but avoid C5b-9-mediated lethality (ATCC 43816). With either scenario, the capsule is likely to be critical. Implicit in the design of bactericidal assays is the assumption that normal human serum contains IgM or IgG subclasses directed against exposed bacterial surface antigens with the capacity to efficiently activate the classical pathway (66); this is certainly the case with much-studied E. coli strains but is less clear with K. pneumoniae. After activation, C5b-9 will engender lethal membrane damage only after disruption of lipid domains on the bacterial surface, resulting in a drastic change to membrane topology and architecture. complement-resistant bacteria may not only mask their cell surface from the initial recognition by the three complement pathways but may also inhibit later stages of the complement pathway by altering their surface configuration in response to envelope stress, preventing membrane insertion and MAC pore formation. Our findings that distinct K. pneumoniae strains can have distinct complement evasion mechanisms, underpinned by dramatically different gene sets, highlights the complexity associated with predicting serum resistance based on genome sequence or single virulence factors—an undertaking which is not yet possible for K. pneumoniae (27). A comprehensive understanding of the basis of complement resistance in Gram-negative bacteria will only be forthcoming when the behavior of such clinically relevant pathogens can be explained.

## MATERIALS AND METHODS

### Construction of the K. pneumoniae B5055 TraDIS library.

The K. pneumoniae B5055 transposon mutant library was constructed by conjugation with E. coli β2163 pDS1028 as described previously (34), with selection of transposon-containing K. pneumoniae B5055 colonies performed at 25°C on LB agar supplemented with 25 μg/ml chloramphenicol. Approximately 600,000 colonies were scraped, pooled, and used as the final B5055 TraDIS library.

### Serum challenge of TraDIS libraries.

Experiments were performed in biological triplicate. TraDIS libraries were grown overnight in 10 ml LB with an inoculum of 10 to 20 μl, which was sufficient to ensure representation of the entire mutant library. Overnight cultures were diluted 1:25, subcultured in 25 ml LB in a 250 ml flask, and grown at 37°C at 180 rpm on an orbital incubator to an optical density at 600 nm (OD_600_) of 1. A 1-ml aliquot of each culture was centrifuged for 2 min at 8,000 × *g* and resuspended in sterile phosphate-buffered saline (PBS). Bacterial suspension (500 μl) was added to 500 μl normal human serum (catalog no. S7023; Sigma) and incubated at 37°C for 90 min. Control reactions were performed in the same way, except that serum was heat-inactivated at 56°C for 30 min prior to use. Following incubation, serum reactions were centrifuged, the pellets suspended in 10 ml LB, and the surviving bacteria outgrown at 37°C for 2 h.

### DNA extraction and next-generation sequencing.

Genomic DNA (gDNA) was purified by phenol-chloroform extraction; 1 to 2 μg DNA was used for the construction of the TraDIS sequencing libraries as described previously (36). Amplification of transposon junctions was performed using primer FS108 (NTUH-K2044, B5055, and ATCC 43816 libraries) or Tn5tetR_5PCR (RH201207 library). Libraries from the RH201207 strain were sequenced on the Illumina MiSeq platform using the primer Tn5tetR_5Seq. All other libraries were sequenced on the Illumina HiSeq platform using FS107. Sequencing was performed as described previously (36).

### Analysis of TraDIS data.

TraDIS sequencing reads were analyzed using the BioTraDIS pipeline as described previously (36, 67), with the following parameters passed to the bacteria_tradis script: “-v -smalt_y 0.96 -smalt_r -1 -t TAAGAGACAG -mm -1.” Reads and insertion sites were assigned to each gene using a custom script (available at https://github.com/francesca-short/tradis_scripts/blob/master/tradis_insert_sites_FS.py), with reads mapping to the 3′ 10% of the gene ignored. Output samples following treatment with serum or heat-inactivated serum were compared to the input sample and to each other using the tradis_comparison.R script without filtering. Hits were defined as those genes with a log_2_ fold change (log_2_FC) of less than −1 or more than 1 (*q* value < 0.005). Values reported in the manuscript are for serum compared to heat-inactivated serum. A previously generated pangenome (34) from a global collection of 265 K. pneumoniae strains (58) was used to identify orthologs between strains and to classify genes as belonging to the K. pneumoniae core or accessory genome. Where needed, pathway information on specific genes was extracted from BioCyc (46).

### Quantification of capsule.

Capsule production was measured using an assay for uronic acids as described previously (68). Overnight cultures of K. pneumoniae were grown in LB at 37°C, and 500-μl aliquots were used directly in the assay. To examine cell-attached capsule, the 500-μl culture samples were centrifuged at 8,000 × *g* for 2 min, and cell pellets were then suspended in 500 μl fresh LB medium prior to uronic acid quantification. A standard curve of glucuronic acid (Sigma-Aldrich) was used to calculate uronic acid concentrations.

### Serum survival assays.

Bacteria were grown overnight in LB, subcultured 1:100 in fresh LB medium, and grown to the late exponential phase (OD_600_ = 1). Cultures were then washed once in PBS and diluted 1:100 in sterile phosphate-buffered saline; 50 μl diluted culture was added to 100 μl prewarmed human serum (Sigma) and incubated at 37°C. Samples were taken at set time points, serially diluted, and plated for enumeration of viable bacteria.

### Hypermucoidy assay.

Overnight cultures of the strains of interest carrying pBAD33-derived Lpp expression plasmids (see Table S1 in the supplemental material) were grown in LB supplemented with 25 μg/ml chloramphenicol, and subcultured for 5 h in LB plus 0.1% l-Ara to induce vector expression. Cultures were centrifuged at 1,000 × *g* for 5 min, and hypermucoidy was expressed as a ratio of OD_600_ of the supernatant/OD_600_ of the original culture.

### Construction of mutants.

Clean single-gene deletion mutants in K. pneumoniae were constructed as described previously (34) using pKNG101Tc-derived allelic exchange vectors introduced by conjugation with E. coli β2163 as the donor strain. Details of the plasmids and oligonucleotides used in mutant construction are in Table S1. Defined transposon insertion mutants of ATCC 43816 and RH201207 were isolated by subjecting the relevant TraDIS library to two rounds of density gradient centrifugation (69). The noncapsulated fraction was grown as single colonies, and mutant locations were identified by randomly primed PCR as described previously (70) using primers FS57 to FS60 together with FS108 and FS109 for ATCC 43816 and FS346 and FS347 for RH201207 (Table S1). Complementation plasmids were constructed using the primers listed in Table S1 and introduced by electroporation.

### Detection of surface-located complement components.

Early mid-logarithmic-phase LB cultures (1 ml) were washed in gelatin veronal buffered saline containing Mg^2+^ and Ca^2+^ (pH 7.35) (GVB^2+^), and incubated in 66% prewarmed (37°C) pooled human serum (MP Biomedicals) for 15, 30, 60, 120, and 180 min (1). Prewarmed, heat-inactivated (56°C; 30 min) human pooled serum served to set the 0-min time point (*T*_0_). Human C3-deficient and C5-deficient serum (Sigma) were used as negative controls. For flow cytometric staining, after incubation the mixtures were washed in PBS and approximately 1 × 10^6^ cells were stained. C3b binding was detected with a mouse monoclonal allophycocyanin (APC) anti-C3b/iC3b antibody (BioLegend) (4 μl per 10^6^ cells), and C5b-9 formation was detected by indirectly staining cells with 8 μg/ml mouse anti-C5b-9 antibody [aE11] (abcam) as the primary antibody and 2.5 μg/ml Alexa Fluor 488 goat anti-mouse IgG H+L (abcam) as the secondary antibody. After 20 min of incubation at room temperature (RT), mixtures were washed and suspended in 200 μl PBS. Samples were acquired using a MACSQuant instrument (Miltenyi Biotec) within 60 min. Approximately 40,000 cell events were collected. Flow cytometry data analysis was carried out using FlowJo 10 Software. GraphPad 7.05 software was used for graph design and statistical analysis.

For microscopy, samples of early mid-logarithmic-phase LB cultures (equivalent to 1 ml at an OD_600_ of 0.5) were washed in GVB^2+^ and incubated with serum for 0, 5, or 15 min. Cells were then washed with PBS, separated into two aliquots, and stained with either 10 μg/ml mouse monoclonal APC anti-C3b/iC3b antibody (BioLegend) or 10 μg/ml mouse anti-C5b-9 antibody [aE11] (abcam) followed by 10 μg/ml Alexa Fluor 488 goat anti-mouse IgG H+L (abcam) for 10 min at RT. Cells were washed with PBS following each staining step, resuspended in PBS, and mounted on 1% PBS agarose pads for imaging. Highly inclined and laminated optical sheet (HILO) microscopy was performed using the Nanoimager S Mark II (Oxford Nanoimaging [ONI]) equipped with 473-nm/300-mW (10%) and 640-nm/300-mW (7%) lasers, dual emission channel split at 560 nm, 100× oil-immersion objective (numerical aperture [NA], 1.49; Olympus) and an ORCA-Flash4.0 v3 complementary metal-oxide-semiconductor CMOS camera (Hamamatsu). Images were acquired at an illumination angle of 51° with 100-ms exposures for >40 frames and processed using FIJI software (71). In brief, transillumination images were generated as an average of 10 frames (total of 1 s of exposure) while fluorescence images were processed by averaging 40 images (total of 4 s of exposure). Brightness and contrast of images in Fig. 6 and 7 are normalized.

### Measurement of cytoplasmic marker release by the B5055 Δ*rfaH* mutant.

Bacteria containing pFLS21 (Table S1), a pDiGc (72) derivative that expresses GFP from the constitutive *rpsM* promoter, were grown to the early log-phase in LB medium and washed once in PBS. A 250-μl aliquot of undiluted cell suspension was combined with 500 μl serum or heat-inactivated serum and incubated at 37°C. The total GFP fluorescence of a 100-μl sample and the fluorescence of 100 μl of supernatant following centrifugation at 8,000 × *g* for 2 min were measured in a Pherastar fluorimeter at set time points following incubation. Values were calculated as percent supernatant fluorescence intensity/total fluorescence intensity, with background signal from 66% human serum with PBS subtracted.

### Statistical analysis.

TraDIS comparisons were conducted using EdgeR as implemented in the BioTraDIS pipeline, for which the statistical approaches have been described in detail. The Benjamini-Hochberg correction for multiple testing was applied.

All quantitative experiments were performed in biological triplicate, with the exception of those shown in Fig. S2B (*n* = 2, 7 technical replicates) and Fig. S4B (*n* = 2) in the supplemental material. All graphs show mean ± 1 standard deviation (SD), and statistical significance is indicated throughout as follows: *, *P* < 0.05; **, *P* < 0.01; ***, *P* < 0.001; ****, *P* < 0.0001. Serum survival data were compared between bacterial strains by two-factor repeated measures analysis of variance (ANOVA) on log_10_-transformed bacterial viable counts with Huynh and Feldt correction. Where the ANOVA indicated a significant time × strain interaction, viability at *t* = 180 was compared by one-way ANOVA with Dunnett’s test for multiple comparisons. Uronic acid quantification and hypermucoidy data were compared between strains by one-way ANOVA on untransformed data followed by Dunnett’s *post hoc* test to compare multiple strains to a single reference or by the Tukey-Kramer test for all-against-all comparisons. Complement binding time series data were tested for significance by two-factor repeated-measures ANOVA on untransformed data, followed by Fisher’s protected least significant difference (LSD) test to compare mutant to wild-type at individual time points.

### Data availability.

The TraDIS sequencing data generated for this study have been deposited in the European Nucleotide Archive (ENA) under project number PRJEB20200. Sample accession numbers are provided in Table S2 in the supplemental material.

## Supplementary Material

Supplemental file 1

Supplemental file 2

Supplemental file 3

Supplemental file 4
