# Supplementary material for: Genomic Profiling Reveals Distinct Routes To Complement Resistance in Klebsiella pneumoniae
Source: Infect Immun. 2020 Jul 21;88(8):e00043-20. doi: 10.1128/IAI.00043-20 (PMC7375759; doi:10.1128/IAI.00043-20)
Supplement: Supplemental file 4 [file IAI.00043-20-s0004.pdf]

Figures

Fig S1 TraDIS results at *K. pneumoniae* capsule loci

Fig S2 Further validation of TraDIS hits

Fig S3 Flow cytometry-based analysis of C3b-positive cell populations

Fig S4 Flow cytometry-based analysis of C5b-9-positive cell populations, and cytoplasmic marker release from B5055 Δ*rfaH*

Tables

Included in this document

Table S1 Strains, plasmids and primers used in this study

Separate documents

Table S2 Sequencing data summary

Table S3 TraDIS full results for four *K. pneumoniae* strains

Table S4 Genes identified as complement resistance determinants by TraDIS

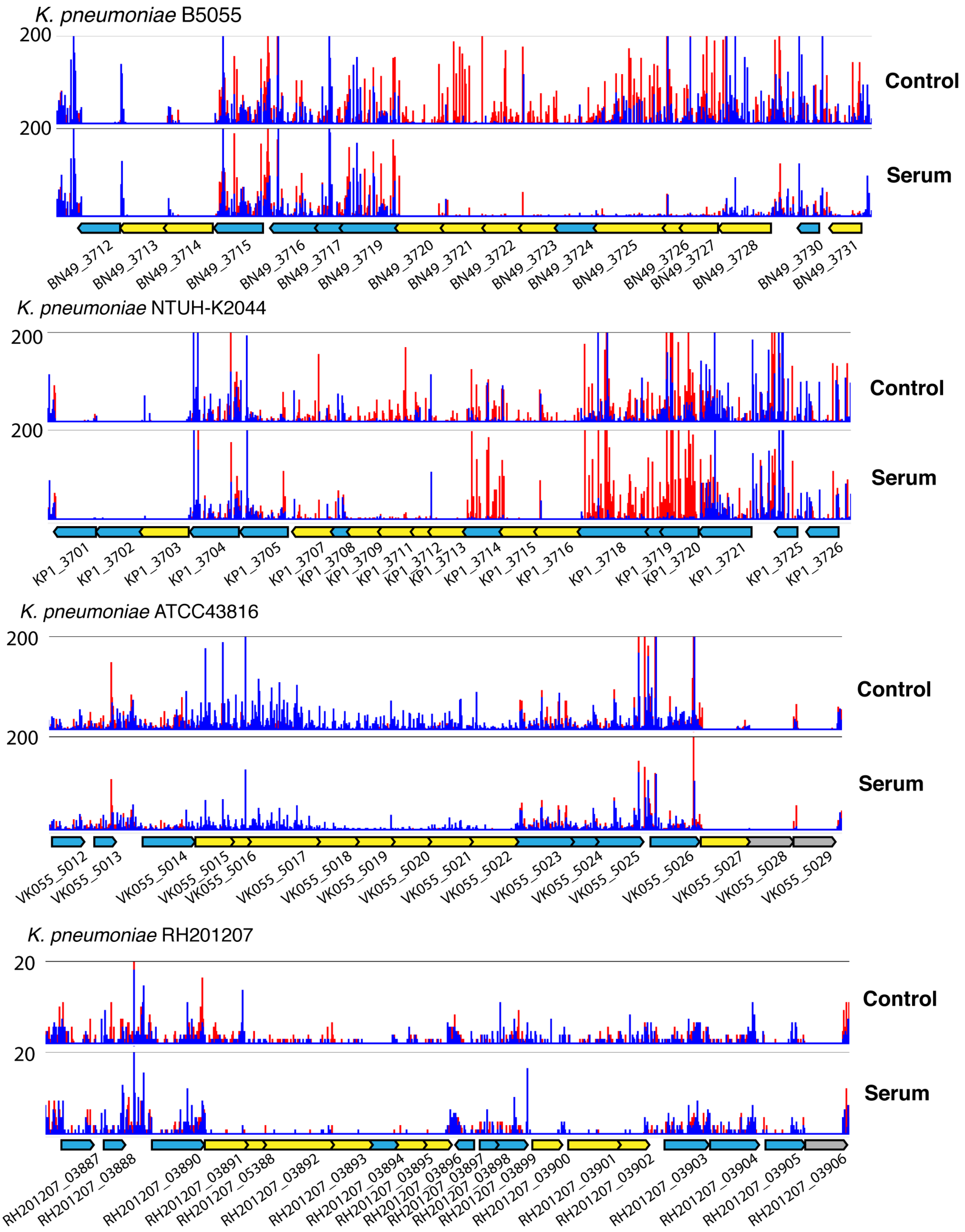

Fig S1 TraDIS results at *K. pneumoniae* capsule loci Insertion plots for TraDIS sequencing at the capsule locus of each strain. Read numbers correspond to the height of the vertical bars, which are coloured blue for plus strand and red for minus strand, respectively. Genes are coloured yellow for hits, grey for essential, and blue for not hits.

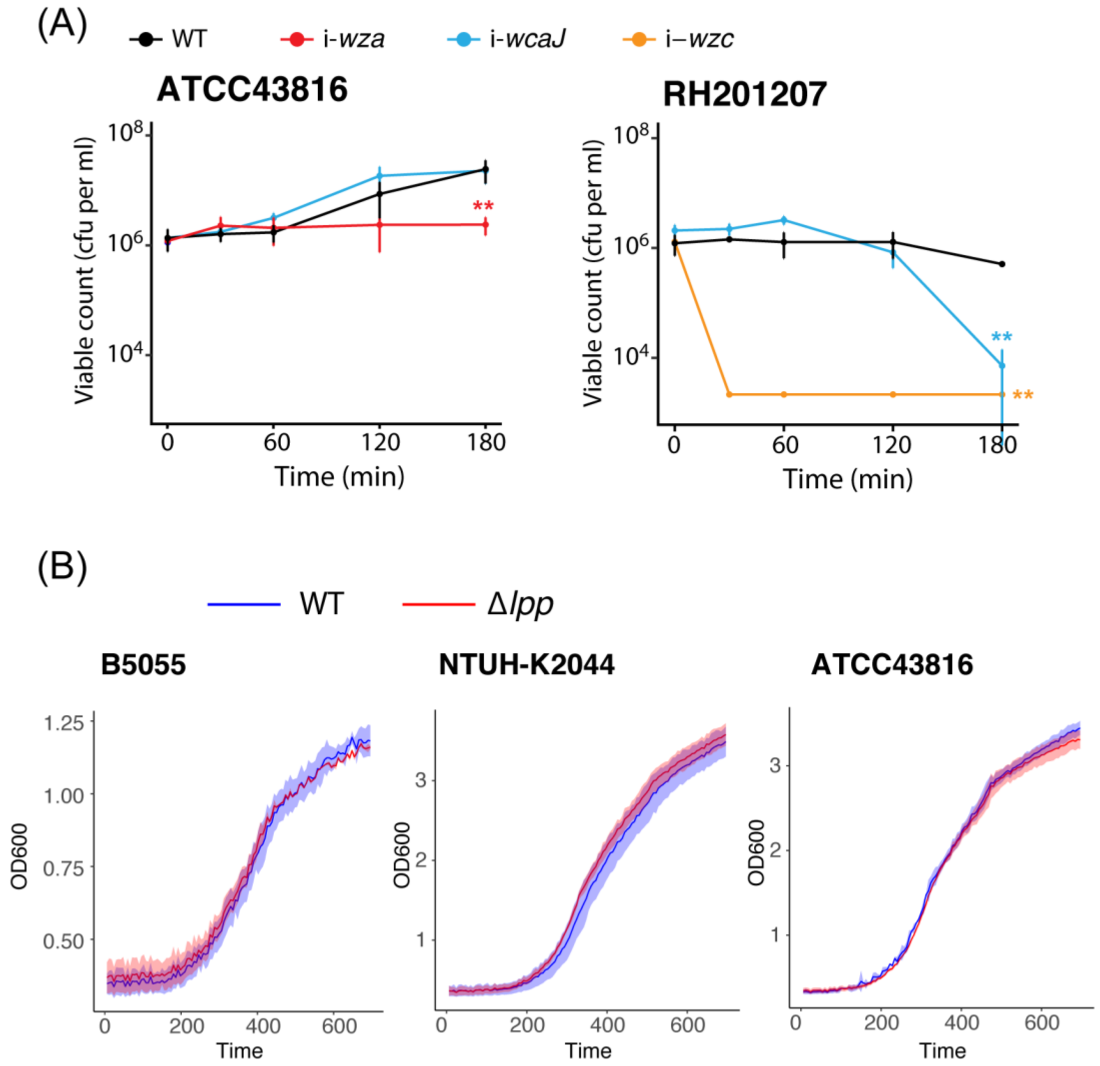

Fig S2 Further validation of TraDIS hits (A) Serum killing assays with additional capsule locus mutants of ATCC43816 (n = 5) and RH201207 (n = 3). Exponential phase cells were incubated with 66% human serum and survival monitored over 3 hours. Statistical significance as determined by two-factor repeated measures ANOVA was $p<0.0001$ for both ATCC43816 and RH201207 (note that these data are derived from the same set of experiments as that shown in Fig 4, and were analysed together). Strain-wise differences relative to wild type at t=180 were determined by one-way ANOVA followed by Dunnett's test. **$p<0.01$ (B) Growth of *K. pneumoniae* Δ*lpp* mutants (n = 2, 7 technical replicates each). Strains were grown with shaking at 37°C in a 96-well plate (200µl/well) in a Pherastar plate reader, and OD600 measured every 6 minutes. Growth of WT and Δ*lpp* strains was compared by two-factor repeated measures ANOVA and was not found to be significant.

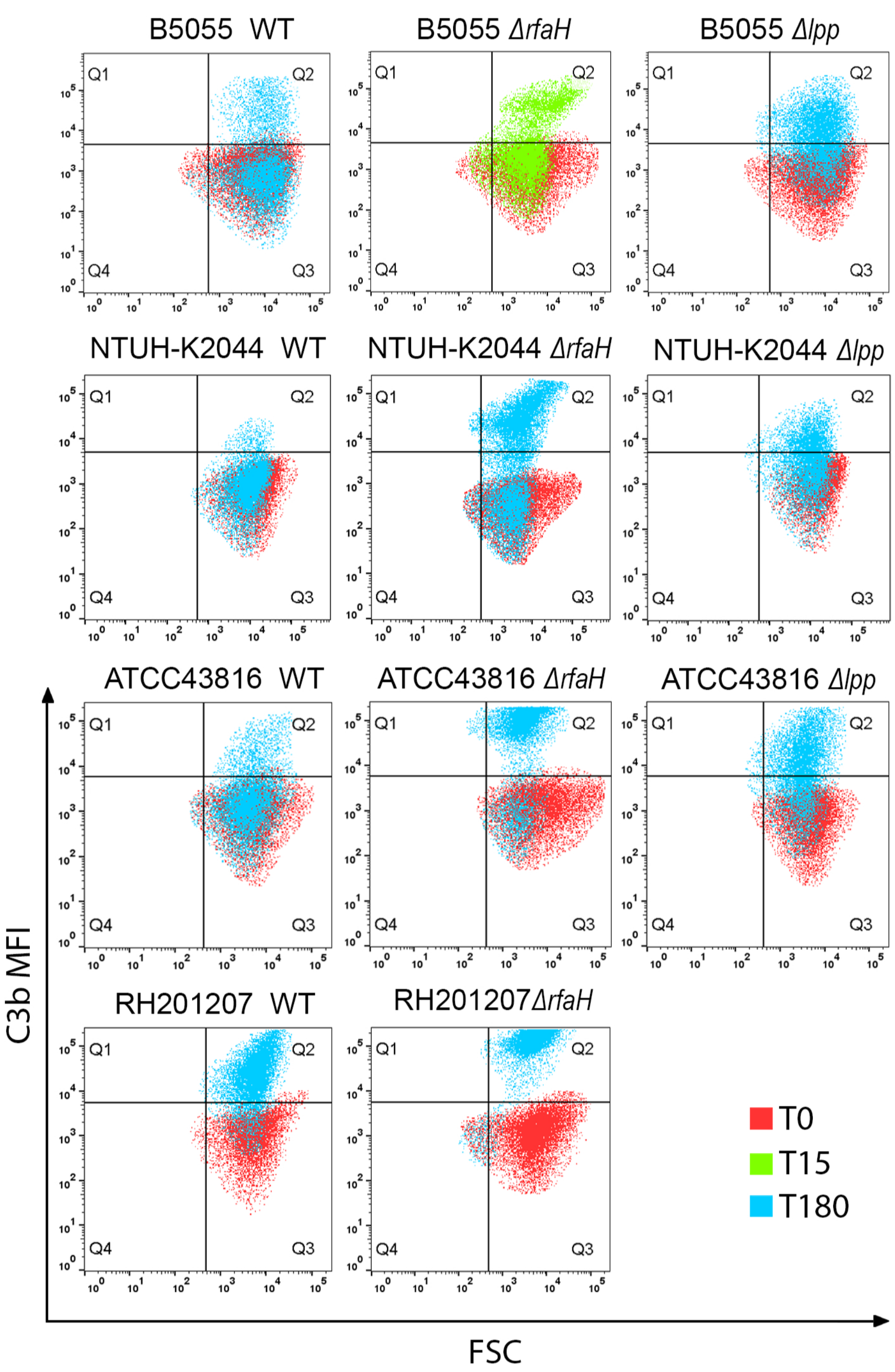

Fig S3 Flow cytometry-based analysis of C3b-positive cell populations Flow cytometry overlay plots illustrate the increase in C3b levels on the bacterial cell surface after 15 min (T15) or 180 min (T180) incubation in human pooled serum at 37°C, compared to T0. C3b positive populations are showed in Q2 quadrants. Data for the four *K. pneumoniae* wildtypes and Δ*rfaH* and Δ*lpp* mutants.

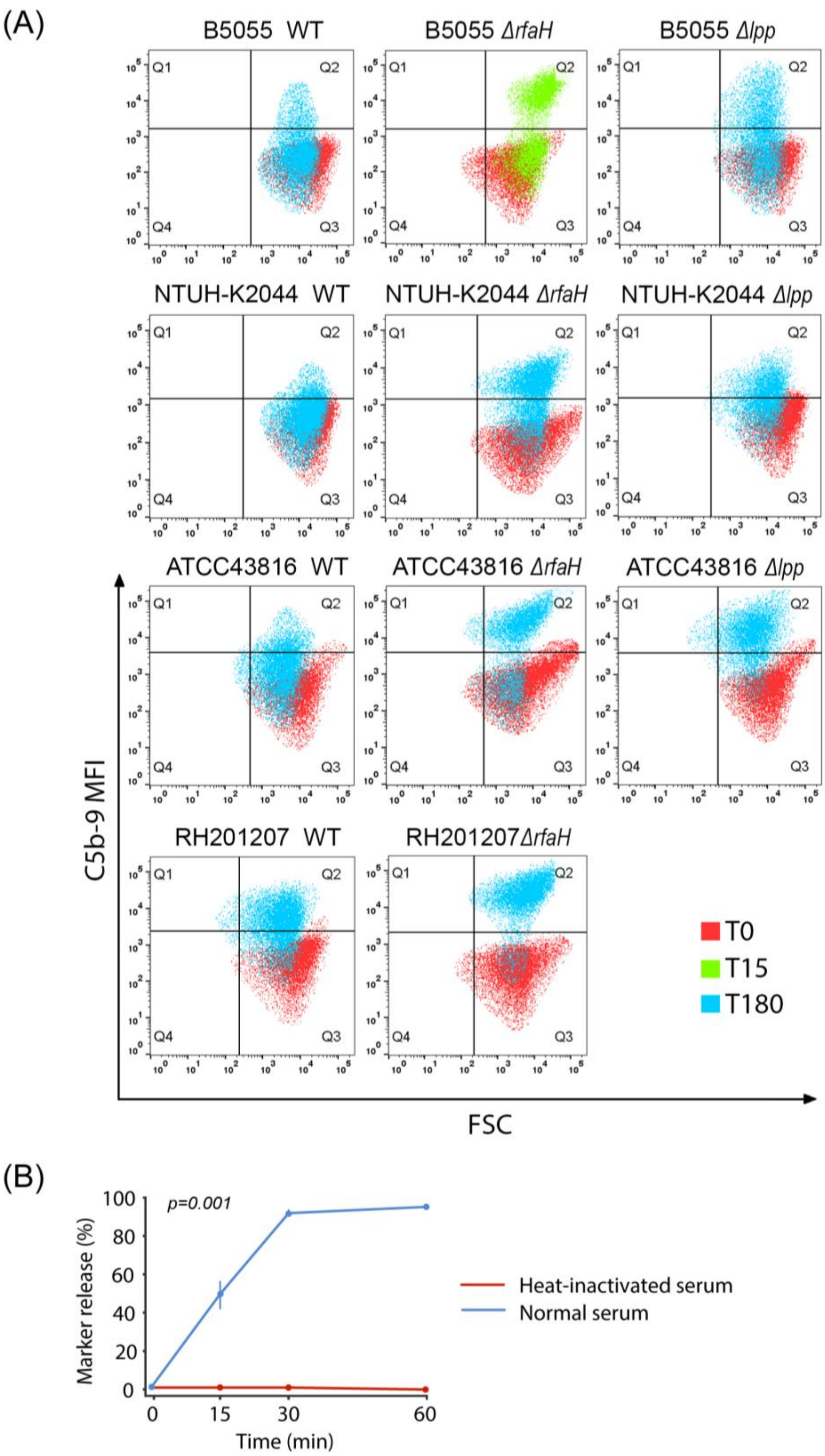

Fig S4 (A) Flow cytometry-based analysis of C5b-9-positive cell populations. Flow cytometry overlay plots illustrate the increase in C5b-9 formation on the bacterial cell surface after 15 min (T15) or 180 min (T180) incubation in human pooled serum at 37°C, compared to T0. C5b-9 positive populations are showed in Q2 quadrants. Data for the four *K. pneumoniae* wildtypes and Δ*rfaH* and Δ*lpp* mutants. (B) Release of cytoplasmic GFP from B5055 Δ*rfaH* carrying the pFLS21 fluorescence marker

vector following incubation in normal or heat-inactivated human serum (n = 2). Results were compared by two-way repeated measures ANOVA.

Table S1 Strains, plasmids and primers used in this study

Bacterial strains

Strain	Description/genotype	Source
Klebsiella pneumoniae ATCC 43816	Hypervirulent, ST493, K-type 2, commonly used in mouse studies	American Type Culture Collection; [1]
Klebsiella pneumoniae NTUH-K2044	Hypervirulent, liver abscess isolate, ST23, K-type 1 reference strain	Jin-Town Wang, National Taiwan University Hospital; [2]
Klebsiella pneumoniae B5055	Mouse-virulent sequence type 66, K-type 2 reference strain	[3]
Klebsiella pneumoniae RH201207	Colistin-resistant UK gut isolate, ST258, K-type 106	Public Health England; [4]
Escherichia coli β2163	F^- RP4-2-Tc::Mu Δ*dapA*::*(erm-pir)*	[5]
K. pneumoniae ATCC43816	Δ*lpp*	This study
K. pneumoniae ATCC43816	Δ*rfaH*	[6]
K. pneumoniae ATCC43816	Tn-*wza*	This study
K. pneumoniae ATCC43816	Tn-*wcaJ*	This study
K. pneumoniae B5055	Δ*lpp*	This study
K. pneumoniae B5055	Δ*rfaH*	This study
K. pneumoniae NTUH-K2044	Δ*lpp*	This study
K. pneumoniae NTUH-K2044	Δ*rfaH*	[6]
K. pneumoniae RH201207	Δ*rfaH*	This study
K. pneumoniae RH201207	Tn5-*wza*	This study
K. pneumoniae RH201207	Tn5-*wzc*	This study

Plasmids

Name	Description	Source
pDS1028	Transposon delivery vector, Tn5-Cm^R *ori*6K, pRL27-derived	[7]
pKNG101-Tc	Allelic exchange vector, Tc^R	[5]
pBAD33	Overexpression vector, Ara-inducible, Cm^R	[8]
pFLS16	pKNG101-Tc derivative, ATCC43816 *rfaH* flanking region	[6]
pFLS21	pDiGc derivative containing Tc^R cassette from	
pFLS31	pKNG101-Tc derivative, ATCC43816/B5055 *lpp* flanking region	This study
pFLS33	pKNG101-Tc derivative, NTUH *lpp* flanking region, constructed with FS342-345	This study
pFLS34	pKNG101-Tc derivative, RH201207 *rfaH* flanking region, constructed with FS136-FS139	This study
pFLS37	pBAD33 derivative, ATCC43816 *lpp* gene, no promoter	This study
pFLS38	pBAD33 derivative, ATCC43816 *lpp* gene ΔK78, no promoter	This study
pMJD82	pACYC184 derivative, NTUH-K2044 *rfaH* gene with native promoter	This study

Primers

Name	Sequence 5' – 3'	Description
FS57	gaccacacgtcgactagtgcnnnnnnnnnnagag	Random-prime PCR
FS58	gaccacacgtcgactagtgcnnnnnnnnnnacgcc	Random-prime PCR
FS59	gaccacacgtcgactagtgcnnnnnnnnnngatac	Random-prime PCR
FS60	gaccacacgtcgactagtgc	Random-prime PCR
FS108	aatgatacggcgaccaccgagatctacaccaggaacacttaacggctgacatgg	pDS1028 Tn 1
FS109	agcctctcaaagcaattttgagtgaca	pDS1028 Tn 2
FS136	tgtgactagtagcggcgcggcatct	RfaH KO Fwd
FS137	tgacattaacggcgtaatgactcattccacttc	RfaH KO Rev overlap
FS138	tgtgactagtgacggtagcagctgga	RfaH KO Rev
FS139	gtggaatgagtcattacgccgttaatgtcaa	RfaH KO Fwd overlap
FS342	gctactagtcgcgctgtgcgcaaatac	Lpp KO Fwd
FS343	tagagggtattaatagagttctggtaataaaaatgg	Lpp KO overlap 1
FS344	ttattaccagaactctattaataccctctagat	Lpp KO overlap 2
FS345	gctactagtccggccggaccgaacaa	Lpp KO Rev
FS335	ctctggtaccctcgcggggcgcttttt	Lpp into pBAD33 Fwd, KpnI
FS336	ctctaagcttttacttacggtaagagtgagc	Lpp into pBAD33 Rev, HindIII
FS337	ctctaagcttttaacggtaagagtgagcctg	LppΔC-terminal Lysine into pBAD33 Rev, HindIII
FS346	cctgatcagggttgagatgtgtataagagacag	RH201207 Tn primer 1
FS347	attgtaggcgccgccctatacctt	RH201207 Tn primer 2
MJD112	ccggatccggtaacggcgcgatgctgtaattttgttaaattag	RfaH Fwd
MJD113	ccgtcgacattagattttctggaagtcggtatttttaac	RfaH Rev

1. Broberg, C.A., Wu, W., Cavalcoli, J.D., Miller, V.L., Bachman, M.A. 2014. Complete Genome Sequence of *Klebsiella pneumoniae* Strain ATCC 43816 KPPR1, a Rifampin-Resistant Mutant Commonly Used in Animal, Genetic, and Molecular Biology Studies. Genome Announcements. 2 (5), e00924-14-e00924-14

2. Wu, K.M. et al. 2009. Genome sequencing and comparative analysis of *Klebsiella pneumoniae* NTUH-K2044, a strain causing liver abscess and meningitis. *Journal of Bacteriology*. 191 (14), 4492–4501

3. Nassif, X., Sansonetti, P.J. 1986. Correlation of the virulence of *Klebsiella pneumoniae* K1 and K2 with the presence of a plasmid encoding aerobactin. Infection and immunity. 54 (3), 603–608

4. Jana, B. et al. 2017. The secondary resistome of multidrug-resistant *Klebsiella pneumoniae.* Scientific Reports. 7, 42483

5. Demarre, G., Guérout, A.M., Matsumoto-Mashimo, C., Rowe-Magnus, D.A., Marlière, P., Mazel, D. 2005. A new family of mobilizable suicide plasmids based on broad host range R388 plasmid (IncW) and RP4 plasmid (IncPα) conjugative machineries and their cognate *Escherichia coli* host strains. Research in Microbiology. 156 (2), 245–255

6. Dorman, M.J., Feltwell, T., Goulding, D.A., Parkhill, J., Short, F.L. 2018. The Capsule Regulatory Network of *Klebsiella pneumoniae* Defined by density-TraDISort. mBio. 9 (6), doi: 10.1128/mBio.01863-18

7. Monson, R. et al. 2015. A Plasmid-Transposon Hybrid Mutagenesis System Effective in a Broad Range of Enterobacteria. Frontiers in Microbiology. 6, 1442

8. Guzman, L.M., Belin, D., Carson, M.J., Beckwith, J. 1995. Tight regulation, modulation, and high-level expression by vectors containing the arabinose PBAD promoter. Journal of bacteriology. 177, 4121–4130
